# Identification of Functionally Distinct Na-HCO_3_ Co-Transporters in Colon

**DOI:** 10.1371/journal.pone.0062864

**Published:** 2013-05-14

**Authors:** Christian Barmeyer, Jeff Huaqing Ye, Carol Soroka, Peter Geibel, Lukas M. Hingsammer, Laurence Weitgasser, Danny Atway, John P. Geibel, Henry J. Binder, Vazhaikkurichi M. Rajendran

**Affiliations:** 1 Department of Internal Medicine, Yale University, New Haven, Connecticut, United States of America; 2 Department of Surgery, Yale University, New Haven, Connecticut, United States of America; 3 Department of Cellular and Molecular Physiology, Yale University, New Haven, Connecticut, United States of America; 4 Department of Biochemistry and Microbiology, West Virginia University School of Medicine, Morgantown, West Virginia, United States of America; Universidade Federal do Rio de Janeiro, Brazil

## Abstract

Na-HCO_3_ cotransport (NBC) regulates intracellular pH (pHi) and HCO_3_ secretion in rat colon. NBC has been characterized as a 5,5′-diisothiocyanato-2-2′-stilbene (DIDS)-sensitive transporter in several tissues, while the colonic NBC is sensitive to both amiloride and DIDS. In addition, the colonic NBC has been identified as critical for pHi regulation as it is activated by intravesicular acid pH. Molecular studies have identified several characteristically distinct NBC isoforms [i.e. electrogenic (NBCe) and electroneutral (NBCn)] that exhibit tissue specific expression. This study was initiated to establish the molecular identity and specific function of NBC isoforms in rat colon. Northern blot and reverse transcriptase PCR (RT-PCR) analyses revealed that electrogenic NBCe1B or NBCe1C (NBCe1B/C) isoform is predominantly expressed in proximal colon, while electroneutral NBCn1C or NBCn1D (NBCn1C/D) is expressed in both proximal and distal colon. Functional analyses revealed that amiloride-insensitive, electrogenic, pH gradient-dependent NBC activity is present only in basolateral membranes of proximal colon. In contrast, amiloride-sensitive, electroneutral, [H^+^]-dependent NBC activity is present in both proximal and distal colon. Both electrogenic and electroneutral NBC activities are saturable processes with an apparent Km for Na of 7.3 and 4.3 mM, respectively; and are DIDS-sensitive with apparent Ki of 8.9 and 263.8 µM, respectively. In addition to Na-H exchanger isoform-1 (NHE1), pHi acidification is regulated by a HCO_3_-dependent mechanism that is HOE694-insensitive in colonic crypt glands. We conclude from these data that electroneutral, amiloride-sensitive NBC is encoded by NBCn1C/D and is present in both proximal and distal colon, while NBCe1B/C encodes electrogenic, amiloride-insensitive Na-HCO_3_ cotransport in proximal colon. We also conclude that NBCn1C/D regulates HCO_3_-dependent HOE694-insensitive Na-HCO_3_ cotransport and plays a critical role in pHi regulation in colonic epithelial cells.

## Introduction

Na-HCO_3_ cotransporters (NBC) are critical for regulating intracellular pH (pHi), HCO_3_ absorption and HCO_3_ secretion [1], [2], [3], [4], [5], [6]. NBC have been classified as electroneutral (NBCn) and electrogenic (NBCe) that mediate Na and HCO_3_ cotransport with a stoichiometry of 1∶1 and 1:>1, respectively (Table-1) [1], [4], [6]. Both NBCn and NBCe have been shown responsible for HCO_3_ movement and pHi regulation in both epithelial and nonepithelial cells [1], [4], [6]. In general, NBC have been characterized as a DIDS (5,5′-diisothiocyanato-2-2′-stilbene)-sensitive (and amiloride-insensitive) transporter in several tissues [1], [4], [6]. However, we demonstrated a novel NBC that is sensitive to both amiloride and DIDS in basolateral membrane vesicles (BLMV) of rat distal colon [7]. Although both NBC and Na-H exchange functions are present in colonic BLMV, NBC has been identified as critical for pHi regulation since acid intravesicular pH activated the former but not the latter [8].

**Table 1:** Splice variants of NBCe and NBCn isoforms and their alternative names.

Isoforms	Variants	Also known as
NBCe	NBCe1A	kNBC [1], [4], [6]
	NBCe1B	pNBC [9], hhNBC [13]
	NBCe1C	bNBC [27]
NBCn	NBCn1A	NBC3 [14]
	NBCn1B	[1], [12]
	NBCn1C	[1], [12]
	NBCn1D	[1], [12]

Molecular studies have isolated several splice variants of NBCe (i.e., NBCe1A, NBCe1B and NBCe1C) and NBCn (NBCn1A, NBCn1B, NBCn1C and NBCn1D) isoforms that exhibit tissue-specific expression patterns [9], [10], [11], [12], [13], [14], [15], [16]. A recent study has shown NBCn1, but not NBCe2 as major pHi regulator in mouse duodenum [17]. However, the molecular identity and the specific function of colonic NBC remain unknown. Studies were, therefore, initiated to identify whether the characteristically distinct colonic NBC is encoded by any of the existing NBC specific transcripts or by one or more totally distinct isoforms. The results presented in this study indicate that the colonic Na-HCO_3_ cotransport activities are encoded by NBCn- and NBCe-like proteins that are expressed in a tissue-specific pattern, as only NBCn-like transcripts are localized in distal colon, while both NBCn-like and NBCe-like transcripts are present in proximal colon. In addition, this study also demonstrates that HOE694 (a specific NHE1-inhibitor)-insensitive NBC plays a major role in pHi regulation.

## Methods

### Ethics Statement

The rats (Sprague-Dawley, 201–225 g) were given an anesthesia using intraperitoneal (i.p) injection of pentobarbital (40–50 mg/kg body wt). Following removal of the colon, the animals were euthanized by injecting 100 mg/kg pentobarbital. The experimental protocols used in this study were approved by the West Virginia University Institutional Animal Care and Use Committee (IACUC #11-0801).

### Northern Blot Analyses

Total RNA from proximal and distal colonic epithelial cells, whole brain, heart and kidney were isolated using TRIzol Reagent (Invitrogen, Carlsbad, CA). mRNA was purified using Oligotex mRNA Midi Kit (Qiagen, Valencia, CA). Northern blot analyses were performed by standard molecular technique, as described previously [18]. In brief, 5 µg mRNA electrophoresed on 1% agarose gel was transferred to a nylon membrane and fixed using Stratalinker (Stratagene, CA). Following a 1–2 h prehybridization period in 50% formamide containing 20% dextransulfate, 2 M NaCl and 1% SDS, the blots were hybridized with the indicated cDNA probes that were labeled using dCT^32^P and the random primer labeling kit (Roche, Mannheim, Germany). Overnight hybridized blots were washed with 2×SSC (sodium chloride, sodium citrate) and 0.1% SDS for 30 min at 45°C. The blots were exposed to Hyper film MP (Amersham Pharmacia Biotech, Little Chalfont, UK) that were incubated at −80°C and were developed after 15 h. The blots were stripped by boiling in 0.1×SSC and 0.5% SDS and used on more than one occasion for hybridization with different probes. Densitometry was performed using Multi-Analyst Software (Bio-Rad, Hercules, CA).

### Reverse Transcriptase-Polymerase Chain Reaction (RT-PCR)

NBCe1A-, NBCe1B/C- and NBCn1-specific segments were amplified by RT-PCR using mRNA from heart, brain, kidney, proximal colon and distal colon. Primers were designed from published sequences (Table 2). RT-PCR was performed using mRNA and OneStep RT-PCR Kit (Qiagen, Valencia, CA). The veracity of RT-PCR products was confirmed by sequencing (Keck Foundation, Yale University, New Haven, CT).

**Table 2:** Gene specific primers used for RT-PCR analyses: bNBC1 (brain NBC1), kNBC (kidney NBC1), NBC2 (NBCn-1b) and NBC3 (NBCn1-d) isoform specific primers were designed from published sequences.

Gene	Primer	Position	Accession number
NBCe1A	Sense: 5′-GGCACAGAGAGAGGAGGCTT-3′ Antisense: 5-TGTCTTCCCAATGTCAGCCAG-3′	17–36 593–613	AF027362
NBCe1B	Sense: 5′-ACTGGAGGAGCGACGGAAG-3′ Antisense: 5′-TGTCTTCCCAATGTCAGCCAG-3′	27–45 730–750	AF210250
NBCn1	Sense 5′-ATCTACCTCCGCTATGTCC-3′ Antisense: 5′-ACTCACAGGCTTTTCAGGGC-3′	3424–3442 3881–3900	AF080106

### Immunofluorescence Studies

Rat proximal and distal colon were flushed with 0.9% saline to remove fecal contents and then rapidly frozen in liquid nitrogen-cooled Freon. Five to ten micron frozen sections were prepared and placed on poly-lysine coated microscope slides. Tissue sections were permeabilized and fixed in acetone at –20°C for 10 minutes, air-dried and rehydrated in PBS. Non-specific sites were blocked with 1% BSA in PBS-0.05% Triton X100. Primary antibodies to NBCe1 [19] and NBCn1 [20] (kindly provided by Dr. Walter F. Boron Yale University, New Haven, Connecticut) were diluted in the blocking buffer and incubated on the tissue for 2 hrs at room temperature. Primary antibodies were subsequently detected with Alexa 594 anti-rabbit IgG (Molecular Probes, Eugene, OR). Microscopy was performed on a Zeiss 510 LSM (Thornwood, NJ) confocal microscope, and the images were processed using Adobe PhotoShop.

### Basolateral Membrane Vesicle (BLMV) Preparation

BLMV were isolated from both proximal and distal colon of normal rats. BLMV were prepared by the sucrose-density gradient and differential centrifugation method, as described previously [7]. In brief, colonic segments harvested from anesthetized rats were filled with 4 mM Hepes-Tris buffer (pH 7.4) containing 5 mM EDTA and 0.5 mM dithiothreitol (DTT). Following 30 min incubation in the same solution the colonic sacs were emptied and the mucosa was scraped using glass slides. The mucosa resuspended in 10 mM Tris-HCl buffer (pH 7.4) containing 250 mM sucrose was homogenized at full speed for 40 strokes using loose fit Teflon homogenizers (Potter-Elvhjem). The homogenates loaded onto continuous (13%–30%) sucrose gradient were isokinetically centrifuged (SW 40.1 rotor, Beckman L7-525 ultracentrifuge) for 7 min at 29,000 rpm. The Na,K-ATPase-rich fractions (top 2–5 ml fractions) from continuous sucrose-gradients were collected and homogenized with 10 strokes at full speed. The homogenates were layered onto continuous-discontinuous sucrose (12%–15%/35% and 55%) gradients and centrifuged for 2 hrs. Membrane fractions collected at the junction between 12%–15% and 35% sucrose were diluted with deionized water (10 times) and centrifuged at 50,000 rpm (Ti 50.1 rotor, Beckman L7-525 ultracentrifuge). Final BLMV pellets were resuspended in appropriate volume of 50 mM MES-Tris buffer (pH 5.5) containing 150 mM K-gluconate and stored at −80°C until used. All solutions used were ice cold, and all preparations were performed at 4°C. Membrane purity was periodically assessed by 10–12 fold enrichment of Na,K-ATPase activity compared to homogenate. Na,K-ATPase activity was measured by the method of Forbush et al [21], as described earlier [22]. Protein assay was performed using the Lowry method [23].

### Uptake Studies

Outward proton and inward HCO_3_ (proton/HCO_3_) gradient-driven and intravesicular positive membrane potential-dependent HCO_3_ gradient-driven ^22^Na (Amersham, Chicago, IL) uptake in BLMV was performed by a rapid filtration technique, as described previously [7]. **Proton/HCO_3_ gradient-driven ^22^Na uptake.** BLMV loaded with 150 mM K-gluconate and 50 mM MES-Tris buffer (pH 5.5) were incubated at room temperature for 6 sec. in medium containing 150 mM KHCO_3_, 0.1 mM ^22^Na and 50 mM HEPES-Tris buffer (pH 7.5). **Membrane potential-dependent HCO_3_ gradient-driven ^22^Na uptake.** BLMV loaded with 150 mM NMG-gluconate and 50 mM HEPES-Tris buffer (pH 7.5) were incubated at room temperature for 6 sec in medium containing 150 mM KHCO_3_, 0.1 mM^22^Na, 50 mM HEPES-Tris buffer (pH 7.5) and 25 µM valinomycin. Uptake was arrested with ice-cold stop solution containing 150 mM K-gluconate and 50 mM HEPES-Tris buffer (pH 7.5) and BLMV were filtered on 0.4 µm filter and washed with 4 ml stop solution. ^22^Na trapped in BLMV collected on filters was dissolved in Optifluor and counted in Beckman counter and used for calculation.

### Crypt Gland Isolation and pHi Studies

**Figure 1:**
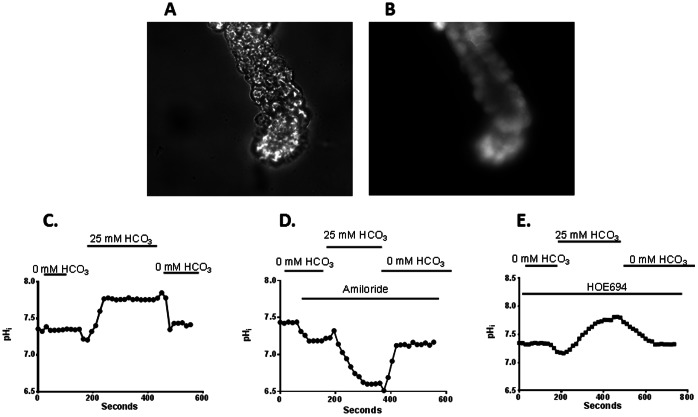
Effect of extracellular HCO_3_ in the presence and absence of HOE694 on intracellular pH change in crypt glands from proximal colon. Images of a crypt gland before [**A**] and after BCECF loading [**B**]. The BCECF loaded crypts were excited at 490 nm and 440 nm and the emission signal was monitored at 535 nm to derive intracellular pH (pHi). [**C**] Under basal condition, bath HCO_3_ increased pHi, while the increased pHi was completely reversed by HCO_3_ removal. [**D**] In the presence of amiloride, bath HCO_3_ reduced the pHi, while the reduced pHi was partially reversed by HCO_3_ removal. [**E**] In the presence of HOE694, bath HCO_3_ increased pHi, while the increased pHi was completely reversed by HCO_3_ removal. Typical tracing presented is representative of observations in 4 different crypts from 5 different rats.

Colonic crypt glands were isolated by the divalent chelation method of Lomax et al [24], as described previously [25]. In brief, distal and/or proximal colon removed from anaesthetized rats filled with HEPES buffer (in mM: 115 NaCl, 5 KCl, 1.2 CaCl_2_, 1.2 MgCl_2_, 2 NaH_2_PO_4_, 32.2 Hepes and 10 glucose; pH: 7.4) were placed in HEPES buffer containing 5 mM EDTA and incubated at 37°C for15 min. The dispersed crypt glands were precipitated by centrifugation at 200–250 rpm (SorvallRC5B, SS34 rotor) for 1 min and the crypts were resuspended in HEPES buffer. Crypt glands were placed on cover-slips coated with the biological adhesive Cell Tak attached to a JG-23 perfusion chamber (Warner Instruments, Hamden, CT). The crypts were then perfused with a HEPES buffer containing BCECF-AM and incubated in the darkness for 30 min. The perfusion chamber was mounted onto the stage of an inverted Olympus IX50 microscope. The chamber was maintained at 37°C using a thermostat feedback system attached to a continuous chamber perfusion system for constant laminar flow. Following loading the chamber was perfused for 5 min. at 37°C to remove any deesterified dye on the surface of the crypt. The cell incorporated BCECF was excited at 490 nm and 440 nm and the emission signal was monitored at 530 nm (Figures 1A and 1B). Initial studies examined the effect of bath HCO_3_ on pHi in the presence and absence of amiloride and HOE694. Under basal condition, bath (i.e., basolateral) HCO_3_ increased pHi, an indication for the presence of HCO_3_-dependent H^+^ extrusion and/or HCO_3_ uptake process on the basolateral membranes of proximal crypt glands (Figure 1C). In the absence of HCO_3_, bath amiloride slightly decreased pHi, an indication for basolateral NHE (i.e., NHE1) mediated H^+^ extrusion in crypts (Figure 1D). In the continued presence of amiloride, bath HCO_3_ substantially reduced the pHi, while the HCO_3_-generated acid pHi partially recovered following bath HCO_3_ removal (Figure 1D). These observations indicate that amiloride-sensitive H^+^ extrussion is regulated by both HCO_3_-dependent and HCO_3_-independent pathways that might be mediated by amiloride-sensitive NBC and NHE1, respectively. To distinguish NBC-dependent H^+^ extrusion from NHE1, the effect of bath HCO_3_ on pHi was also examined in the presence of bath HOE694 (a NHE1 specific inhibitor) [26]. In contrast to amiloride, bath HCO_3_ in the presence of HOE694 increased the pHi, an indication for the presence of HCO_3_-dependent H^+^ extrusion process on the basolateral membranes (Figure 1E). These observations indicate that both HOE694-sensitive/HCO_3_-independent (i.e., NHE1) and HOE694-insensitive/HCO_3_-dependent (i.e., NBC) H^+^ extrusion processes are present on the basolateral membranes of proximal crypt glands.

In additional studies, crypt glands were sequentially perfused with HEPES buffer, HEPES buffer containing HOE694 (1 µM) and HEPES buffer containing HCO_3_ (25 mM) plus HOE694 (1 µM). Crypts were also sequentially perfused with HEPES buffer containing HCO_3_ plus HOE694, HEPES containing HOE694 and HEPES buffer containing HCO_3_ plus HOE694. Before termination of the experiments with high-K^+^ calibration the crypts were exposed to HEPES buffer containing HOE694. The data recorded as the ratio between 490/440 nm was converted to intracellular pH (pH_i_) after calibration with the high K buffer (in mM: 105 KCl, 1.2 CaCl_2_, 1.2 MgCl_2_, Hepes 32.2, NMDG-32.2 and mannitol 5; pH 7.0) and Nigericin. Osmolality of all solutions used was adjusted to 295–300 mOsm.

### Statistical Analysis

Data presented represent mean ± SE of triplicate assays of three different membrane preparations for uptake studies, and 3 crypts from three different distal colon. ANOVA statistical analyses were performed using the SPSS program. *p*<0.05 were considered statistically significant.

## Results

**Figure 2:**
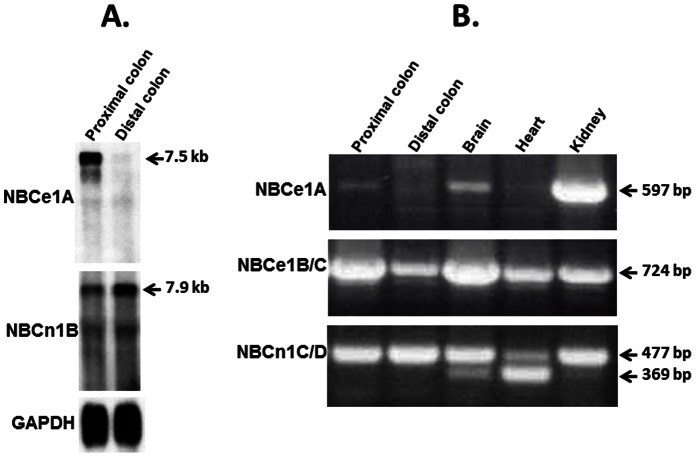
Northern blot and RT-PCR analyses of mRNA from rat colon. [**A**] Northern blot analyses indicate that NBCe1A (7.5 kb)-like mRNA is predominantly expressed in proximal colon, while NBCn1B (7.9 kb)-like mRNA is expressed in both proximal and distal colon. Full length NBCe1A and NBCn1A cDNAs were used as probe. The blots were also probed with a GAPDH cDNA fragment. Results presented are from an individual blot that was stripped and probed sequentially with each probe (NBCe1A, NBCn1A and GAPDH). [**B**] RT-PCR analyses indicate that NBCe1B/C and NBCn1C/D specific transcripts are predominantly expressed in epithelial cells from proximal and distal colon. NBC isoform specific fragments were amplified by RT-PCR using isoform specific primers (Table 2) and mRNA from proximal colon, distal colon, brain, heart and kidney. RT-PCR products were separated on 1% agarose gels and stained with ethidium bromide. cDNA size markers are indicated on the right side.

To identify whether any existing NBC-like isoforms are expressed in the rat colon, northern blot analyses of proximal and distal colon mRNA were performed using full length NBCe1A (also known as kNBC1) and NBCn1A (also known as NBC3) cDNA as probes [1], [14], [15]. As shown in Figure 2, NBCe1A cDNA hybridized with a 7.5 kb transcript in proximal colon, but barely identified any mRNA in distal colon (Figure 2A). In contrast, the NBCn1 probe hybridized with a 7.9 kb transcript in both proximal colon and distal colon (Figure 2A). These observations indicate that both NBCe1- and NBCn1-like mRNAs are expressed in proximal colon, while, in contrast, only NBCn1-like mRNA is identified in distal colon.

In additional studies, RT-PCR analyses were performed to establish the specific NBCe1 and NBCn1 isoforms that are expressed in these colonic segments as at least three splice variants of NBCe1 [NBCe1A (kidney), NBCe1B (pancreas and heart), and NBCe1C (brain)] [9], [13], [15], [27] and four splice variants of NBCn1 [NBCn1A, NBCn1B, NBCn1C and NBCn1D (Table-1), according to the terminology by Choi et al [12] have been reported from various tissues [1], [12], [14]. RT-PCR analyses were performed using mRNA from both proximal and distal colon, and compared to RT-PCR analyses of mRNA from brain, heart and kidney. As shown in Figure 2B, the 725 bp fragment that is common in 5′-region of both NBCe1B and NBCe1C (NBCe1B/C) that is amplified in brain, heart and kidney, is also amplified in both proximal and distal colon. NBCe1B and NBCe1C isoforms differ only at 3′-end [1]. This is in contrast to the northern blot findings which did not reveal any NBCe1 expression in distal colon. A possible explanation is that RT-PCR is more sensitive and can identify even smallest amounts of mRNA in a given tissue. However, there is no experimental support for NBCe1 function in distal colon [8]. In contrast to NBCe1B/C, the NBCe1A-specific fragment, which is predominantly amplified from brain and kidney, is absent in distal colon (Figure 2B). These results demonstrate that either NBCe1B or NBCe1C is likely to be the predominant NBCe1 variant in both proximal and distal colon. However, from these results the presence of a different yet unidentified NBCe1 variant cannot be excluded. The NBCn1-specific 477 bp fragment that is amplified from both proximal and distal colon corresponds to the “B cassette” as seen by Choi et al [12] in NBCn1C/D indicating that either one or both of these or a third yet unidentified isoform containing the “B cassette” is present in rat colon (Figure 2B). The 369 bp fragment amplified in brain and heart corresponds to NBCn1B which is missing the “B cassette”. The NBCn1B specific fragment was not amplified from either proximal or distal colon (data not shown).

**Figure 3:**
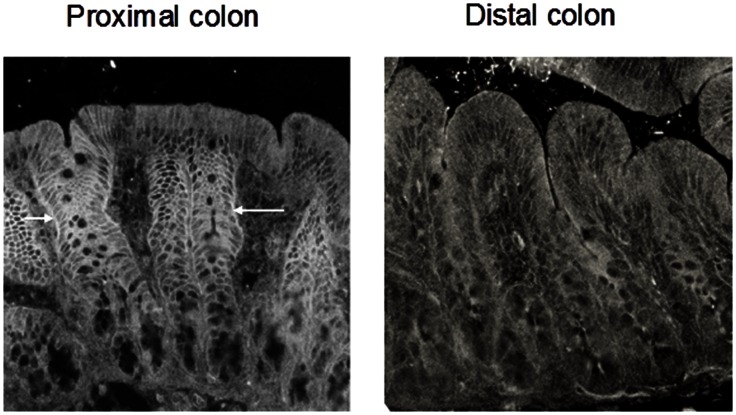
Immunocytochemical localization of NBCe1-like proteins in rat proximal and distal colon. [**A**] Rat proximal colon demonstrates strong basolateral staining for NBCe1 in epithelial cells of the mid-crypt region (arrows) and weaker staining in surface cells. [**B**] Rat distal colon shows no localization of NBCe1 in the epithelial cells. Magnification 40x.

**Figure 4:**
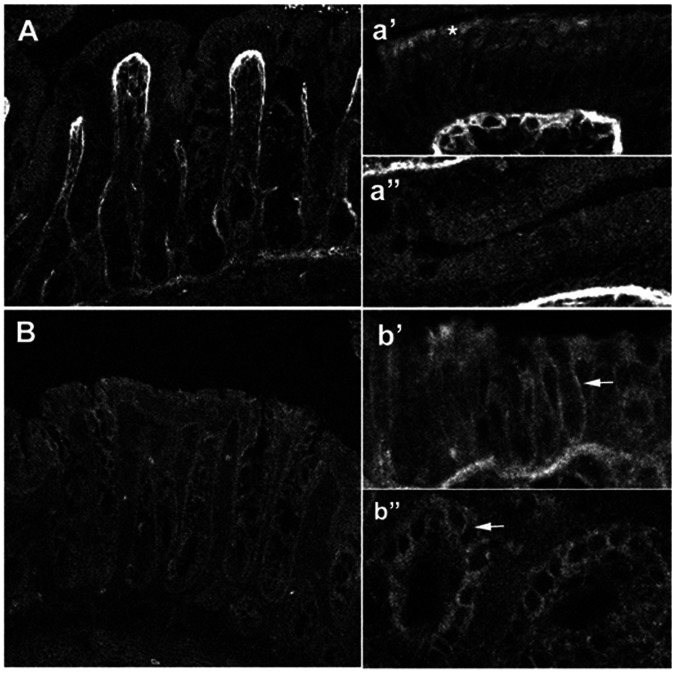
Immunocytochemical localization of NBCn1-like proteins in rat proximal and distal colon. [**A, a’, a”**] Rat proximal colon demonstrates weak epithelial staining for NBCn1 along the crypt to surface axis. The connective tissue underlying the epithelial cells is strongly fluorescent. Higher magnification of the surface cells [**a’**] shows a rather punctuate apical signal [*]. High magnification of epithelial cells in the crypt region (a”) demonstrates a weak diffuse localization. [**B, b’, b”**] Rat distal colon shows stronger staining for NBCn1. High magnifications of surface cells [**b’**] and crypt cells [**b”**] clearly demonstrate basolateral membrane localization [**arrows**]. Smooth muscle cells were also strongly stained (data not shown).

Although northern blot analyses indicated that NBCe-like mRNA expression is not present in distal colon (Figure 2A), RT-PCR analyses provided evidence that NBCe1B/C isoform specific mRNA may be present in distal colon (Figure 2B). Therefore, immunofluorescence studies were performed in both proximal and distal colon using a polyclonal anti-peptide antibody raised against a fusion protein that is common to all NBCe isoforms [19]. As shown in Figure 3, NBCe-like proteins are predominantly localized on the basolateral membranes of mid-crypt regions of proximal colon, but are only weakly present in the surface cells. In contrast, NBCe-like proteins are not localized in epithelial cells of distal colon (Figure 4). These observations are consistent with the northern blot analyses (Figure 2A) that NBCe-like NBC is present only in proximal colon but not in distal colon. The RT-PCR amplification of NBCe1B/C-like transcripts from distal colon (Figure 2B) may be due to either cross contamination of proximal mRNA or nominal expression of NBCe1B/C transcripts in distal colon. Thus, in the proximal colon both NBCe1B/C and NBCn1C/D specific mRNAs are present while in the distal colon NBCn1C/D specific mRNA is primarily expressed.

Immunofluorescence studies were also performed to establish the cell- and membrane-specific localization of NBCn-like proteins in proximal and distal colon. Figure 4 shows that NBCn-like proteins are weakly present in basolateral membranes of both surface and crypt cells in proximal colon (Figures 4A, 4a’, 4a”). At higher magnification NBCn-like proteins are also localized in apical membranes of proximal colon (Figure 4a’). Strong fluorescence is also present in connective tissue underlying the epithelial cells. In contrast, NBCn-like protein expression is stronger in basolateral membranes of both surface (Figure 4b’) and crypt cells (Figure 4b”) of distal colon. NBCn-like proteins are not localized in apical membranes of distal colon (Figure 4B). NBCn-like proteins are also localized in smooth muscle cells (data not shown). It has to be noted here that the human NBCn2A, which has been shown to exhibit ethylisopropylamiloride (EIPA; an amiloride analogue)-sensitive Na-HCO_3_ cotransport activity in an oocyte expression system was originally cloned form skeletal muscle [14]. These observations suggest that NBCn-like isoforms with significant homology may be present in basolateral membranes of both proximal and distal colon.

**Figure 5:**
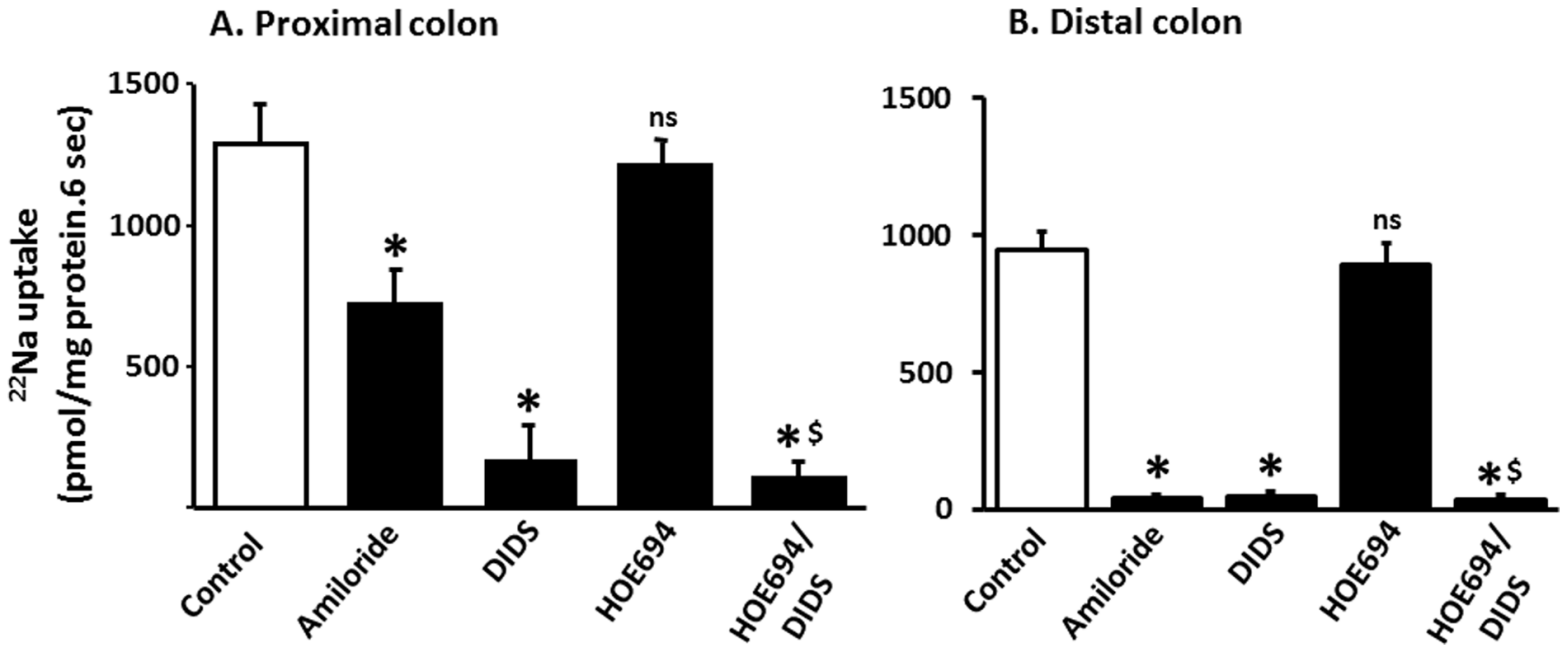
Effect of inhibitors on Na-HCO_3_ cotransport activities in basolateral membrane vesicles from proximal and distal colon. Proton/HCO_3_ gradient-driven ^22^Na uptake (0.1 mM) was measured in the absence (Control) and presence of inhibitors (1 mM amiloride; 1 mM DIDS; 1 µM HOE694) in basolateral membrane vesicles (BLMV) from rat proximal [**A**] and distal [**B**] colon, as described in the method section. Amiloride partially inhibited, while DIDS almost completely inhibited the Na-HCO_3_ cotransport activity in BLMV from proximal colon. Both amiloride and DIDS inhibited the Na-HCO_3_ cotransport activity in BLMV from distal colon. HOE694 did not significantly inhibit Na-HCO_3_ cotransport in BLMV from either proximal or distal colon. *p<0.001 - compared to control; ^$^ *p*<0.001 - compared to HOE694; ns: not significant compared to control.

The immunofluorescence studies establish that both NBCe-like and NBCn-like proteins are present in basolateral membranes of proximal colon, while only NBCn-like proteins are present in basolateral membranes of distal colon (Figures 3 and 4). Therefore, experiments were designed to identify whether functionally distinct Na-HCO_3_ cotransporters are present in basolateral membrane vesicles (BLMV) of colon. In these studies, similar to our prior study with distal colon [7], ^22^Na uptake measured in presence of both outward proton and inward HCO_3_ (proton/HCO_3_) gradients provided evidence for Na-HCO_3_ cotransport in both proximal and distal BLMV (Figure 5). It is important to emphasize that neither the presence nor the absence of Cl affected proton/HCO_3_ gradient-driven ^22^Na uptake in these BLMV (unpublished observations). In initial studies the effect of 1 mM amiloride, 1 mM DIDS and 1 µM HOE694 on proton/HCO_3_ gradient-driven ^22^Na uptake was examined. Similar to our earlier observations, both amiloride and DIDS almost completely inhibited proton/HCO_3_ gradient-driven ^22^Na uptake in distal BLMV (Figure 5B) [7]. In contrast, although DIDS inhibited proton/HCO_3_ gradient-driven ^22^Na uptake by similar magnitude, amiloride only partially (44%) inhibited proton/HCO_3_ gradient-driven ^22^Na uptake in proximal BLMV (Figure 5B). Since amiloride-sensitive ^22^Na uptake could represent Na-H exchange as NHE1 isoform is also present in proximal BLMV [18], the effect of HOE694 was also examined. Since prior studies established HOE694 to inhibit NHE1 with a kinetic constant of 0.16 µM, 50 µM HOE694 was used for complete inhibition of NHE1 function in BLMV [28]. As shown in Figures 5A and 4B, proton/HCO_3_ gradient-driven ^22^Na uptake was not significantly inhibited by HOE694. The HOE694-insensitive proton/HCO_3_ gradient-driven ^22^Na uptake was inhibited by 95% by the addition of DIDS (Figure 5). These observations establish that HOE694-insensitive proton/HCO_3_ gradient-driven ^22^Na uptake occurs via a DIDS-sensitive transport that consists of both amiloride-sensitive and amiloride-insensitive Na-HCO_3_ cotransport processes in the basolateral membranes of proximal colon. These observations that both amiloride-sensitive and amiloride-insensitive components are present in proximal colon but only amiloride-sensitive component is present in distal colon combined with the molecular studies showing the presence of NBCe1B/C and NBCn1C/D in proximal colon but only NBCn1C/D in distal colon raises the possibility that NBCn1C/D encodes the amiloride-sensitive NBC activity, while NBCe1B/C encodes amiloride-insensitive Na-HCO_3_ cotransport activity.

**Figure 6:**
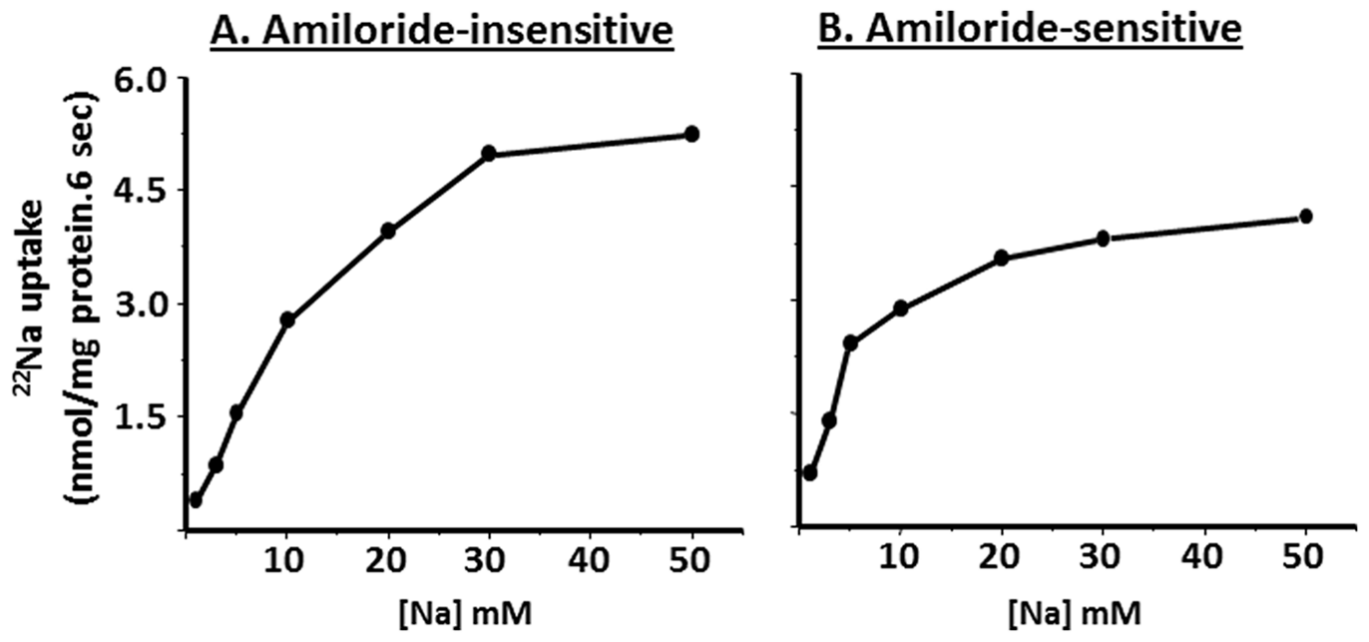
Kinetics of Na-HCO_3_ cotransport activities in basolateral membrane vesicles from proximal colon. Increasing extravesicular Na concentrations (1–50 mM) saturate both amiloride-insensitive and amiloride-sensitive proton/HCO_3_ gradient-driven ^22^Na uptake with apparent Km for Na of 7.3±0.6 and 4.3±0.3 mM, respectively. Proton/HCO_3_ gradient-driven ^22^Na uptake was measured as described in the Method section with increasing extravesicular Na concentrations both in presence and absence of inhibitors (either 1 mM amiloride or 1 µM HOE694). Uptake obtained in presence of amiloride represented amiloride-insensitive proton/HCO_3_ gradient-driven uptake. Amiloride-sensitive uptake (which also includes HOE694 sensitive Na-H exchange) was calculated by subtracting uptake obtained in presence of amiloride from that of uptake in the absence of inhibitors. HOE694-sensitive uptake was calculated by subtracting uptake obtained in presence of HOE694 from that of uptake in the absence of inhibitors. Amiloride-sensitive proton/HCO_3_ gradient-driven uptake was calculated by subtracting HOE694-sensitive uptake from that of amiloride-sensitive uptake. Best fit curves were drawn using Michaelis-Menten equation. The kinetic constants were calculated using a nonlinear regression with the EnzFitter software (Biosoft, Cambridge, UK).

**Figure 7:**
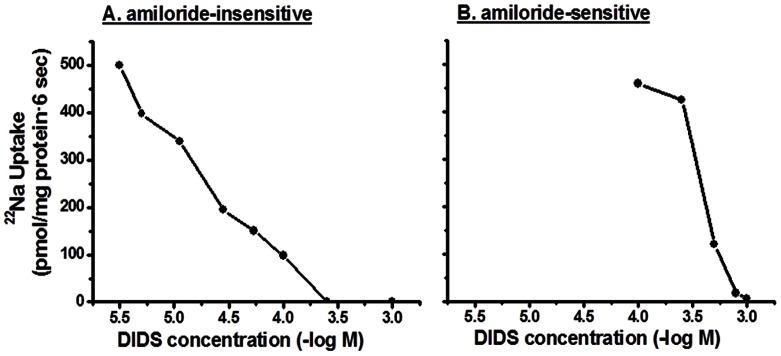
DIDS-inhibition kinetics of Na-HCO_3_ cotransport activities in basolateral membrane vesicles from proximal colon. Increasing DIDS concentrations inhibit both amiloride-insensitive and amiloride-sensitive proton/HCO_3_ gradient-driven ^22^Na uptake with half maximal inhibitory concentration (Ki) of approximately 8.9±1.2 and 263.8±26.4 µM, respectively. Proton/HCO_3_ gradient-driven ^22^Na uptake (0.1 mM) was measured as described in the Method section with increasing extravesicular DIDS concentrations both in presence and absence of inhibitors (either 100 µM EIPA or 1 µM HOE694). Uptake obtained in presence of amiloride represented amiloride-insensitive proton/HCO_3_ gradient-driven uptake. Amiloride-sensitive uptake (which also includes HOE694 sensitive Na-H exchange) was calculated by subtracting uptake obtained in presence of EIPA from that of uptake in the absence of inhibitors. HOE694-sensitive uptake was calculated by subtracting uptake obtained in presence of HOE694 from that of uptake in the absence of inhibitors. Amiloride-sensitive proton/HCO_3_ gradient-driven uptake was calculated by subtracting HOE694-sensitive uptake from that of EIPA-sensitive uptake. The inhibitory constants were calculated using a nonlinear regression with the EnzFitter software (Biosoft, Cambridge, UK).

**Figure 8:**
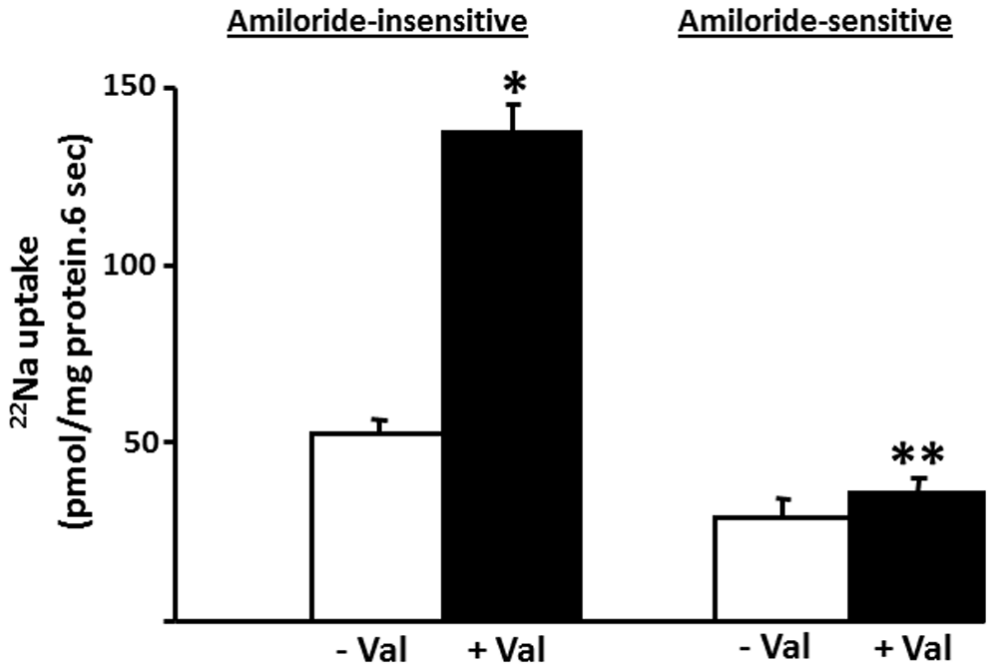
Effect of intravesicular membrane potential on Na-HCO_3_ cotransport activities in basolateral membrane vesicles from proximal colon. Intravesicular positive membrane potential [generated using inward K^+^ gradient and valinomycin (Val)] dependent HCO_3_-gradient-driven ^22^Na (0.1 mM) uptake was measured as described in the Method section both in presence and absence of 1 mM amiloride. Intravesicular positive potential stimulated amiloride-insensitive HCO_3_ gradient-driven ^22^Na uptake. Intravesicular positive potential did not affect amiloride-sensitive Na-HCO_3_ cotransport. Uptake obtained in presence of amiloride represented amiloride-insensitive uptake, while amiloride-sensitive uptake was calculated by subtracting the amiloride-insensitive uptake from that of uptake in the absence of amiloride. *p<0.001 compared to –Val in amiloride-insensitive; **p<0.05 compared to –Val in amiloride-sensitive.

Kinetic studies were performed to distinguish the characteristics of amiloride-sensitive and amiloride-insensitive Na-HCO_3_ cotransport in proximal BLMV. As shown in Figure 6, increasing extra-vesicular Na concentrations (1–50 mM) saturate both amiloride-insensitive and amiloride-sensitive Na-HCO_3_ cotransporters with Km for Na of 7.3±0.6 and 4.3±0.3 mM, respectively. Increasing extravesicular DIDS concentrations inhibit both amiloride-insensitive and amiloride-sensitive Na-HCO_3_ cotransport with apparent half-maximal inhibitory concentrations (Ki) of 8.9±1.2 and 263.8±26.4 µM, respectively (Figure 7). In additional studies, the effect of intravesicular positive membrane potential (generated with an inward K gradient and valinomycin) was examined as electrogenic Na-HCO_3_ cotransporters have also been shown to mediate the movement of two or more HCO_3_ for each Na ion [1], [4], [6]. As shown in Figure 8, intravesicular positive membrane potential substantially stimulated amiloride-insensitive Na-HCO_3_ cotransport in proximal BLMV. In contrast, amiloride-sensitive Na-HCO_3_ cotransport was not altered by the intravesicular positive membrane potential. These observations indicate that amiloride-sensitive Na-HCO_3_ cotransport is electroneutral and relatively less sensitive to DIDS, while amiloride-insensitive Na-HCO_3_ cotransport is electrogenic and highly DIDS-sensitive, and further establish that amiloride-sensitive and amiloride-insensitive Na-HCO_3_ cotransporters are distinct and separate carrier-mediated transport processes in basolateral membranes of rat proximal colon. These functional studies are consistent with the molecular experiments that concluded that in the proximal colon both NBCn1C/D and NBCe1B/C were present, while in the distal colon where NBC activity was predominantly electroneutral and amiloride-sensitive, only NBCn1C/D was identified.

**Figure 9:**
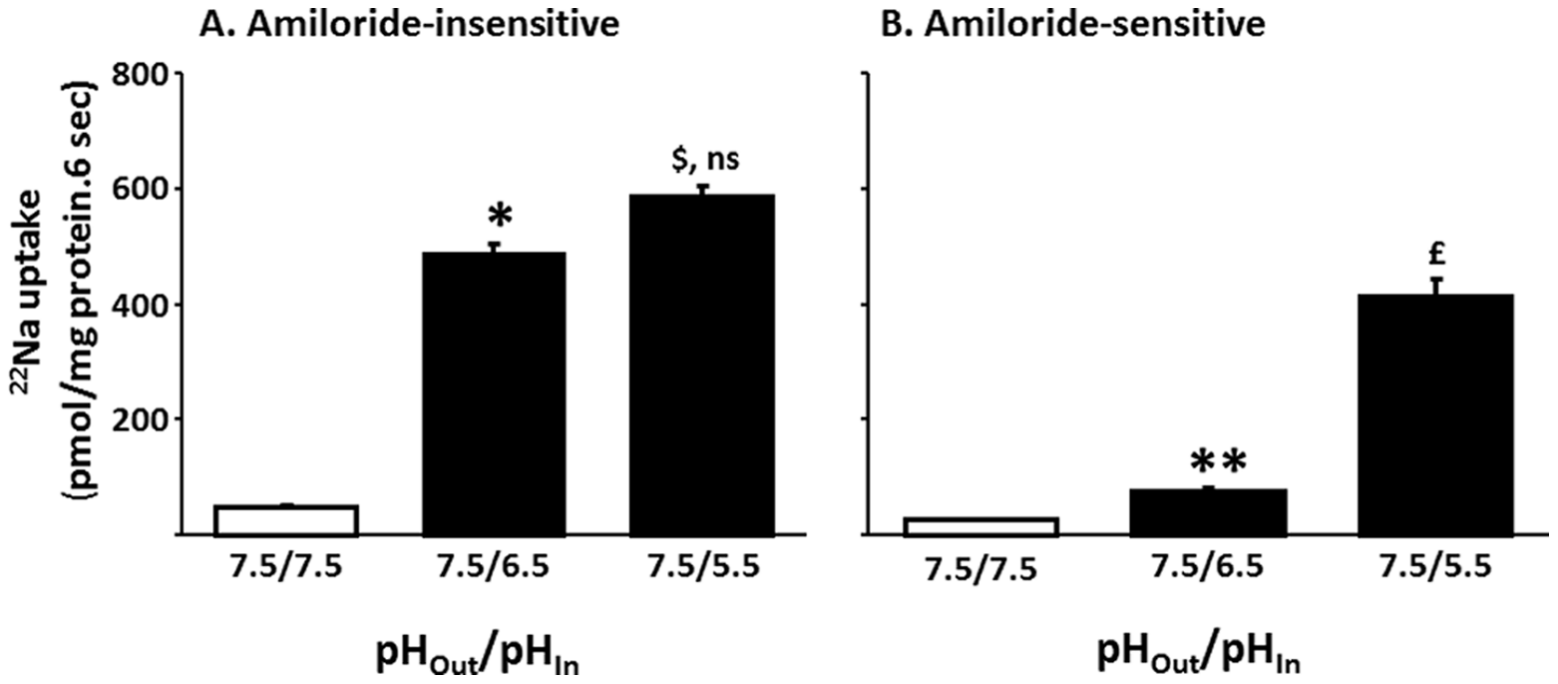
Effect of varying outward acid pH-gradients on Na-HCO_3_ cotransport activities in basolateral membrane vesicles from proximal colon. Proton/HCO_3_ gradient-driven ^22^Na uptake (0.1 mM) was measured as described in the Method section with different extravesicular pH in presence and absence of inhibitors (1 mM amiloride or 1 µM HOE694). Both one-unit (pHo/pHi: 7.5/6.5) and two-unit (pHo/pHi: 7.5/5.5) outward acid pH-gradients stimulated the amiloride-insensitive Na-HCO_3_ cotransport. A two-unit, but not a one-unit outward acid pH gradient stimulated amiloride-sensitive Na-HCO_3_ cotransport. Uptake obtained in presence of amiloride represented amiloride-insensitive proton/HCO_3_ gradient-driven uptake. Amiloride-sensitive uptake (which also includes HOE694 sensitive Na-H exchange) was calculated by subtracting uptake obtained in presence of amiloride from that of uptake in the absence of inhibitors. HOE694-sensitive uptake was calculated by subtracting uptake obtained in presence of HOE694 from that of uptake in the absence of inhibitors. Amiloride-sensitive proton/HCO_3_ gradient-driven uptake was calculated by subtracting HOE694-sensitive uptake from that of amiloride-sensitive uptake. *p<0.02 compared to 7.5/7.5 (pHo/pHi) in amiloride-insensitive; *^$^p*<0.002 compared to 7.5/7.5 (pHo/pHi) in amiloride-insensitive; **p<0.004 compared to 7.5/7.5 (pHo/pHi) in amiloride-sensitive; *p<0.001 compared to 7.5/7.5 (pHo/pHi) in amiloride-sensitive; ns: not significant compared to 7.5/6.5 (pHo/pHi) in amiloride-insensitive.

Studies were designed to establish whether amiloride-insensitive and amiloride-sensitive Na-HCO_3_ cotransport of proximal BLMV are regulated by proton concentration and/or by the magnitude of the pH gradient since amiloride-sensitive Na-HCO_3_ cotransport in rat distal colon is regulated by proton concentration [8]. In these studies, the effect of one- and two-unit outward acid pH gradient with a constant extravesicular pH (pHo/pHi: 7.5/6.5 vs 7.5/5.5), as well as a one-unit pH gradient at different pHs (pHo/pHi: 8.0/7.0 vs 7.0/6.0) was examined. Both amiloride-insensitive and amiloride-sensitive Na-HCO_3_ cotransport activities were minimal in the absence of a pH gradient (Figure 9). Imposition of a one-unit pH gradient significantly (12-fold) enhanced amiloride-insensitive Na-HCO_3_ cotransport, but there was no further enhancement in presence of a two-unit pH gradient (Figure 9). In contrast, amiloride-sensitive Na-HCO_3_ cotransport was only minimally enhanced by a one-unit pH gradient but was substantially (26-fold) stimulated by a two-unit pH gradient (Figure 9). These observations suggest that, although an outward proton gradient is important for maximal activation of both amiloride-insensitive and amiloride-sensitive Na-HCO_3_ cotransporters, amiloride-insensitive activity is maximally stimulated by a ten-fold proton concentration gradient while a hundred-fold proton concentration gradient is required for stimulation of amiloride-sensitive NBC activity.

**Figure 10:**
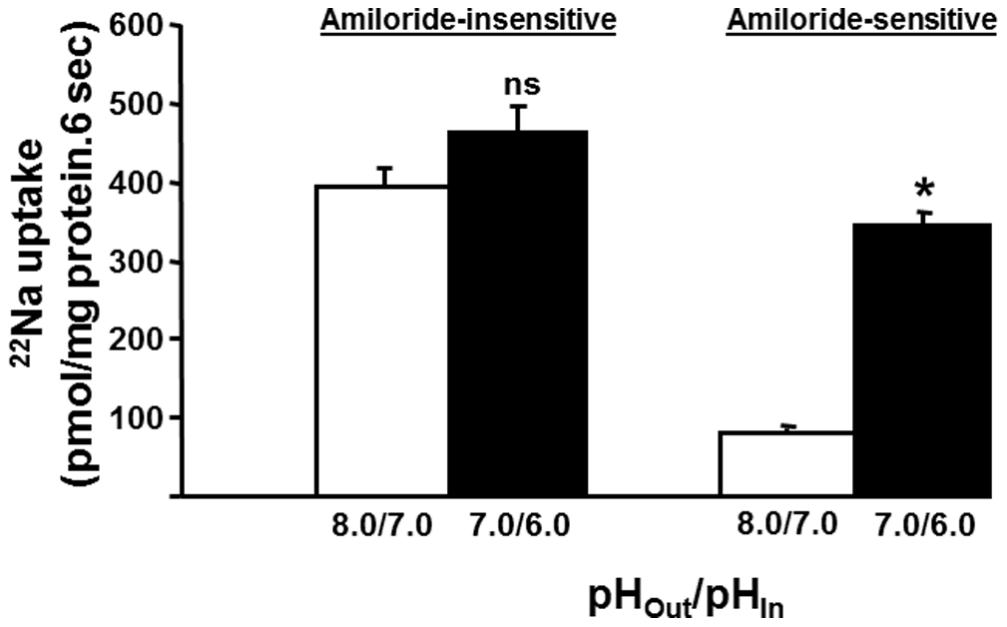
Effect of one-unit pH-gradient with varying proton concentrations on Na-HCO_3_ cotransport activities in basolateral membrane vesicles from proximal colon. Proton/HCO_3_ gradient-driven ^22^Na uptake (0.1 mM) was measured as described in the Method section with one unit acid outward pH gradient at different extravesicular and intravesicular pH in presence and absence of inhibitors (1 mM amiloride or 1 µM HOE694). One-unit outward acid pH-gradients with both low (pHo/pHi: 8.0/7.0) and high (pHo/pHi: 7.0/6.0) proton concentrations stimulated amiloride-insensitive Na-HCO_3_ cotransport, while amiloride-sensitive Na-HCO_3_ cotransport was substantially stimulated only at high proton concentrations. Uptake obtained in presence of amiloride represented amiloride-insensitive proton/HCO_3_ gradient-driven uptake. Amiloride-sensitive uptake (which also includes HOE694 sensitive Na-H exchange) was calculated by subtracting uptake obtained in presence of amiloride from that of uptake in the absence of inhibitors. HOE694-sensitive uptake was calculated by subtracting uptake obtained in presence of HOE694 from that of uptake in the absence of inhibitors. Amiloride-sensitive proton/HCO_3_ gradient-driven uptake was calculated by subtracting HOE694-sensitive uptake from that of amiloride-sensitive uptake. *p<0.001 - compared to 8.0/7.0 (pHo/pHi) in amiloride-sensitive; ns: not significant - compared to 8.0/7.0 (pHo/pHi) in amiloride-insensitive.

Since the results presented in Figure 9 do not distinguish between absolute proton concentration and magnitude of the proton concentration gradient, the effect of a one-unit outward acid pH gradient at different pHs was examined on amiloride-insensitive and amiloride-sensitive Na-HCO_3_ cotransport. As shown in Figure 10, the amiloride-insensitive Na-HCO_3_ cotransport activity which is maximally stimulated by a one-unit pH gradient is identical at two different pHs (i.e., 8.0/7.0 vs 7.0/6.0) indicating that the absolute proton concentration is not critical. In contrast, amiloride-sensitive Na-HCO_3_ cotransport activity is approximately 4-fold higher in presence of a one-unit pH gradient at 7.0/6.0 than in presence of a one-unit pH gradient at 8.0/7.0 (Figure 10). It is also possible that amiloride-sensitive and amiloride-insensitive NBC may reflect different intravesicular and extravesicular pH sensitivity, and that the amiloride-sensitive NBC may be highly sensitive at intravascular pH ≤6.0. The observations that the activation of amiloride-insensitive and amiloride-sensitive Na-HCO_3_ cotransport activities by pH gradients and by proton concentration gradients differ substantially suggest that these two HCO_3_ transporters regulate different cellular functions in rat proximal colon.

**Figure 11:**
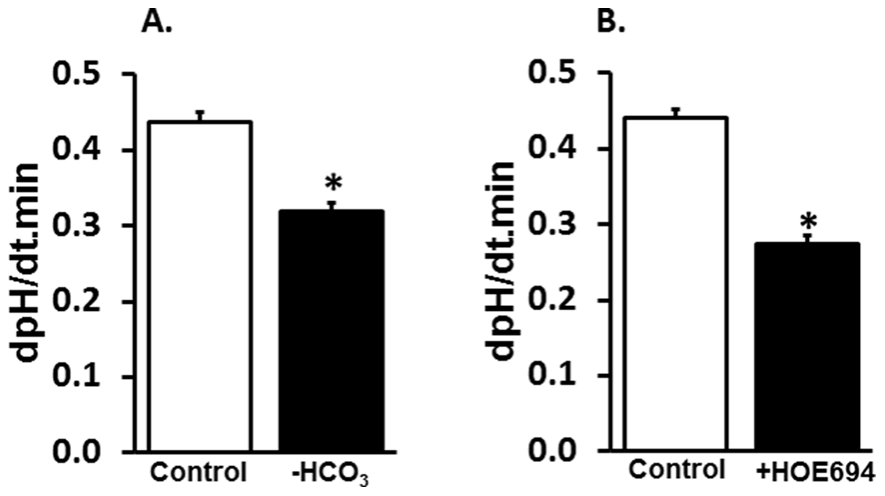
Effect of extracellular HCO_3_ and HOE694 on the rate of intracellular pH (pHi; proton extrusion) in crypt glands from proximal colon. [**A**] Rate of change in pHi was examined in presence (**Control**) and absence (**-HCO_3_**) of extracellular HCO_3_. [**B**] Rate of change in pHi in the presence of extracellular HCO_3_ was examined in presence (**HOE694**) and absence (**Control**) of 1 µM HOE694. *p<0.001 - compared to respective control.

Since amiloride-sensitive Na-HCO_3_ cotransport is regulated by [H^+^]-gradients (Figures 9 and 10), experiments were designed to identify whether amiloride-sensitive Na-HCO_3_ cotransport plays any role in the regulation of pHi in the colon. In this study the role of Na-HCO_3_ cotransport on pHi regulation was determined by measuring pHi in crypt glands isolated from proximal colon (Figures 1A and 1B). Partial pHi acidification (dpHi/dt; control vs –HCO_3_∶ 0.437±0.013 vs 0.320±0.011; *p*<0.001) by bath HCO_3_ removal indicates the presence of HCO_3_-independent H^+^ extrusion pathway in crypt cells from proximal colon (Figure 11A). Since HCO_3_ diffusion would have contributed to the pHi acidification in HCO_3_-free medium, the effect of HOE694 was examined on pHi in presence of bath HCO_3_. Similar to HCO_3_ removal, the NHE1 inhibitor HOE694 also only partially acidified the pHi in crypt cells from proximal colon (dpHi/dt control vs +HOE694∶ 0.440±0.012 vs 0.274±0.011; *p*<0.001) (Figure 11B). These observations indicate that both HCO_3_-dependent (i.e., HOE694-insensitive) and HCO_3_-independent (i.e., HOE694-sensitive) H^+^-extrusion pathways are present in basolateral membranes of crypt cells from proximal colon.

**Figure 12:**
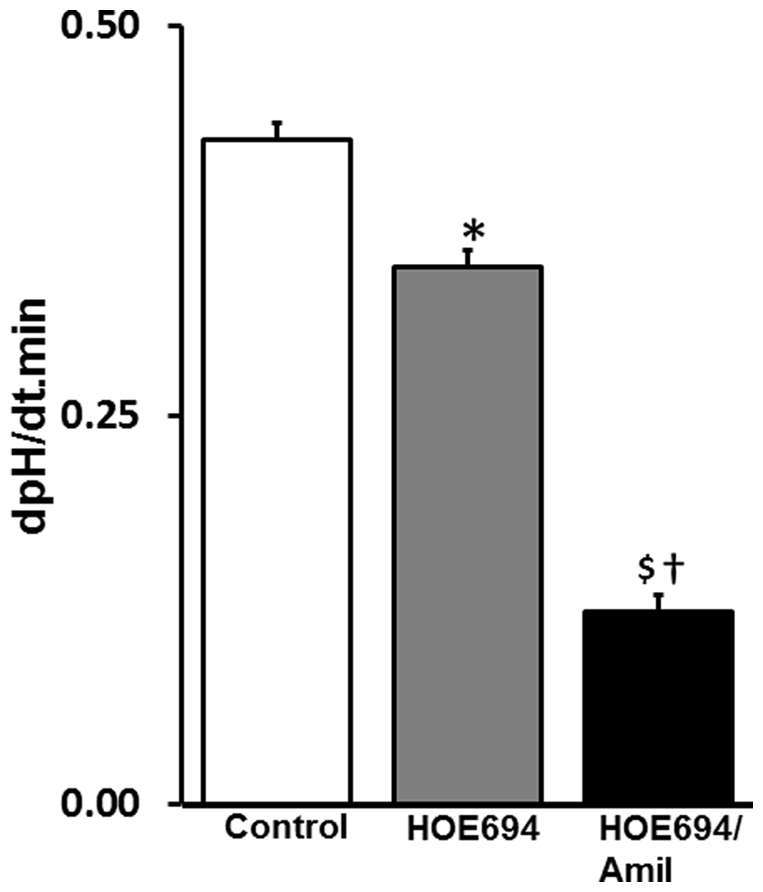
Effect of HOE694 on intracellular pH (pHi; proton extrusion) in crypt gland from distal colon. Rate of change in pHi in presence of extracellular HCO_3_ was examined in the presence (**HOE694**) and absence (**Control**) of 1 µM HOE694. Rate of pHi in the presence of HOE694 was also measured in additional presence of 0.1 mM amiloride (**HOE694/Amil**). *p<0.0003 - compared to control; ^$^ *p*<0.0001– compared to control; ^†^ *p*<0.0001– compared to HOE694.

Studies were also designed to test whether amiloride-sensitive HCO_3_-dependent H^+^-extrusion plays a critical role in pHi regulation. In these studies, pHi regulation in presence of bath HCO_3_ was performed in crypt glands isolated from distal colon, which is devoid of NBCe expression (Figures 2A and 3) and amiloride-insensitive NBC activity [7]. As shown in Figure 12, HOE694, which inhibits NHE1-mediated H^+^-extrusion, only partially but significantly acidified the pHi in crypt cells (dpHi/dt control vs –HOE694∶ 0.427±0.011 vs 0.346±0.011; *p*<0.0003). This observation indicates that, in addition to HOE694-sensitive H^+^-extrusion (i.e., NHE1 mediated H^+^ extrusion), a HOE694-insensitive H^+^-extrusion pathway is also present in crypt from distal colon. Thus, to determine whether NBCn1 mediates the HOE694-insensitive fraction of H^+^ extrusion, the effect of amiloride (0.1 mM) was examined on pHi acidification. In the presence of HOE694, bath amiloride further acidified pHi in crypt cells from distal colon (dpHi/dt HOE694 vs HOE694/amiloride: 0.346±0.011 vs 0.124±0.011; *p*<0.0003). These observations indicate that amiloride-sensitive H^+^-extrusion consists of both HOE694-sensitive (i.e., NHE1-mediated) and HOE694-insensitive (i.e., NBCn mediated) H^+^-extrusion processes in basolateral membranes of crypt cells from distal colon. The substantially higher rate of pHi acidification by amiloride compared to that by HOE694 alone (dpHi/dt HOE694 vs HO694/amiloride: 0.081±0.017 vs 0.222±0.028; *p*<0.001) indicates that amiloride-sensitive H^+^ extrusion mediated via NBCn plays a major role in pHi regulation in colonic crypts.

## Discussion

HCO_3_ secretion is an essential colonic function that is present in both health and diarrhea. HCO_3_ secretion requires intracellular HCO_3_ accumulation as a result of either cell metabolism and/or HCO_3_ uptake across colonocytes’ basolateral membranes and an exit mechanism across their apical membranes. In recent studies, we have identified three distinct mechanisms for HCO_3_ movement across the apical membranes of rat distal colon: 1) Cl-dependent HCO_3_ secretion (i.e., Cl-HCO_3_ exchange); 2) short-chain-fatty acid (SCFA)-dependent HCO_3_ secretion (i.e., SCFA-HCO_3_ exchange); and 3) cAMP-induced HCO_3_ secretion (via anion channels) [29]. In these studies we also have demonstrated that all three of these HCO_3_ secretory processes require the presence of HCO_3_ in the serosal bath solution indicating the importance of a basolateral HCO_3_ uptake process for HCO_3_ secretion [29]. Studies with BLMV have identified both Cl-HCO_3_ exchange and Na-HCO_3_ cotransport processes as possible mechanisms for HCO_3_ uptake across basolateral membranes of rat distal colon [7], [30]. In support of a role for Na-HCO_3_ cotransport in HCO_3_ movement, basolateral Na-HCO_3_ cotransporters have been shown to mediate both HCO_3_ secretion and absorption in renal proximal tubules and pancreatic ducts [1], [2], [31], [32]. However, Na-HCO_3_ cotransport is also important for the regulation of pHi [33]. Thus, molecular studies have isolated several electrogenic and electroneutral Na-HCO_3_ cotransport isoforms which have been shown to be expressed in a tissue-specific pattern [1], [4], [6]. However, although Cl-HCO_3_ exchange has been identified as an anion exchange isoform-2 (AE2) in rat distal colon [30], the molecular identities of Na-HCO_3_ cotransport in the rat colon have not as yet been identified.

Our present northern blot analyses indicate that both NBCe- and NBCn-like transcripts are expressed in proximal colon, while only NBCn- and not NBCe-like mRNAs and proteins are expressed in distal colon (Figures 2, 3 and 4). RT-PCR analyses confirmed that NBCe- and NBCn-like proteins might be encoded by NBCe1B/C- and NBCn1C/D- (or yet unidentified isoforms containing the amplified fragments) specific transcripts in colon, respectively (Figure 2B). It is not known whether these two isoforms mediate identical (or similar) HCO_3_-linked functions or whether these two isoforms regulate two distinct HCO_3_-linked activities.

These studies also established that NBC activities in the proximal and distal colon were not identical in that amiloride-sensitive NBC activity was present in both proximal and distal colon, while amiloride-insensitive activity was only present in the proximal colon (Figure 5) [7]. The observation of both the expression of NBCn specific transcripts and the presence of amiloride-sensitive Na-HCO_3_ cotransport activity in BLMV of proximal and distal colon suggests that NBCn1 transcripts encode amiloride-sensitive Na-HCO_3_ cotransport in colonic basolateral membranes (Figures 2A, 3, 4 and 5) [7]. Human NBCn1A, which is 89–92% identical to the rat NBCn1 isoforms expressed in Xenopus oocytes has been shown to exhibit EIPA-, an amiloride analogue, sensitive Na-HCO_3_ cotransport activity which support the hypothesis that colonic NBCn1 transcripts encode amiloride-sensitive Na-HCO_3_ cotransport activity in both proximal and distal BLMV [14]. On the other hand, the observations that NBCe-specific mRNA and amiloride-insensitive Na-HCO_3_ cotransport activity are present only in BLMV of proximal, but not in distal colon indicate that NBCe1B/C specific Na-HCO_3_ cotransport mediates amiloride-insensitive Na-HCO_3_ cotransport in the basolateral membrane of proximal colon. This conclusion is consistent with other studies that observed decreased cAMP-stimulated HCO_3_ secretion and SITS-sensitive current in the colon of NBC1^−/−^ mice indicating that NBC1 activity is a component of basolateral HCO_3_ uptake during cAMP-stimulated anion secretion in the proximal colon [34] and the involvement of NBCe and NBCn in basolateral electroneutral and electrogenic HCO_3_ transport in proximal duodenal villus cells [35].

Our studies revealed that amiloride-insensitive Na-HCO_3_ cotransport requires a pH gradient, whereas the magnitude of amiloride-sensitive Na-HCO_3_ cotransport is dependent on high proton concentration (Figures 9 and 10). As discussed, NBCe1B/C-like and NBCn1C/D-like transcripts encode amiloride-insensitive and amiloride-sensitive Na-HCO_3_ cotransport functions in rat colon, respectively. Thus, we propose that amiloride-insensitive NBCe1B/C mediates transcellular transport of HCO_3_, while NBCn1C/D regulates pHi homeostasis. The conclusion that NBCe1B/C mediates transcellular transport of HCO_3_ is supported by the demonstrations that: 1) amiloride-insensitive Na-HCO_3_ cotransport activity is predominantly present in proximal, but not in distal colon; and 2) NBCe1B/C-like mRNA is localized in a tissue (proximal colon) specific pattern (Figures 2A and 2B). Rat kidney NBCe has been shown to be activated by intracellular acid pH in a Xenopus oocyte expression system [36] and mouse NBCe has been shown to contribute to pHi regulation in parotid acinar cells [37]. However, there are no studies that characterized the effect of pHi on NBCn isoforms. It should, therefore, be of interest to compare and contrast the pH dependency of NBCe and NBCn isoforms in an expression system to define their specific role.

Our study also demonstrates that a HCO_3_-dependent amiloride-sensitive transporter plays a major role in pHi regulation in epithelial cells of the colonic crypt. This conclusion is supported by the observations that there is a 2.7-fold higher rate of pHi acidification in presence of amiloride than that found in the presence of HOE694 alone (Figure 12). Although both NHE1 and amiloride-sensitive NBC are present, bath amiloride-sensitive H^+^ extrusion (i.e., pHi acidification) has always been presumed to be mediated only by NHE1 [28], [38]. The present study used HOE694 to distinguish amiloride-sensitive NBC (i.e., NBCn1)-mediated H^+^ extrusion from that of NHE1-mediated H^+^ extrusion in colonic crypts. NBCn1 has also been shown as the major pHi regulator in mouse duodenal epithelial cells [17]. In that study, Chen et al have used a NBCn1 knockout mouse to demonstrate the role of NBCn1 mediated pHi neutralization as a defensive mechanism against cellular acidification generated by gastric acid in duodenum [17].

These studies identified two distinct NBC (NBCe1B/C and NBCn1C/D) isoforms in proximal colon, but only one isoform (NBCn1C/D) in distal colon. The transport characteristics of these two isoforms have been established: NBCe1B/C is amiloride-insensitive, highly DIDS-sensitive (Ki: 8.9±1.2 µM), not activated by high proton concentrations, but activated by a proton gradient. In contrast, NBCn1C/D is amiloride-sensitive, less sensitive to DIDS (Ki: 263.8±26.4 µM) and activated by high proton concentrations. HCO_3_ secretion has been identified in both proximal and distal colon [39]. Detailed studies of HCO_3_ secretion in the distal colon demonstrate that basolateral HCO_3_ movement is required but suggest that this uptake process involves NKCC (Na-K-2Cl cotransport) and not NBC. Although parallel studies of HCO_3_ secretion have not been performed in the proximal colon, we speculate that NBCe1B/C may be responsible for HCO_3_ uptake and that the molecular mechanism of HCO_3_ secretion in proximal and distal colon differs. In addition, studies are in progress to clone NBCe1B/C-like and NBCn1C/D-like cDNAs from rat colon to establish whether these isoforms exhibit amiloride-insensitive and amiloride-sensitive Na-HCO_3_ cotransport in an in vitro expression system, respectively.
